# A rare entity of Primary Ewing sarcoma in kidney

**DOI:** 10.1186/s12893-020-00948-9

**Published:** 2020-11-11

**Authors:** Li Cheng, Yujie Xu, Hong Song, Houbao Huang, Dong Zhuo

**Affiliations:** 1 Department of Urology, Anhui Province, The First Affiliated Hospital of Wannan Medical College, Wuhu, People’s Republic of China; 2 Department of Pathology, Anhui Province, The First Affiliated Hospital of Wannan Medical College, Wuhu, People’s Republic of China

**Keywords:** Renal ewing sarcoma (RES), Primitive neuroectodermal tumor (PNET), Nephrectomy, Chemotherapy

## Abstract

**Background:**

Ewing sarcoma (ES) or primitive neuroectodermal tumors (PNET) represents a spectrum of poorly differentiated and aggressive malignancies. It rarely arises from the kidney and accounts for less than 1% of renal mass. Given the uncharacteristic clinical symptoms and imaging features, renal Ewing sarcoma (RES) is often diagnosed by postoperative pathology.

**Case presentation:**

Herein, we depicted a case of RES, which was administrated in our institution by chief complaints of intermittent left plank pain and palpable abdominal mass. We demonstrated the aggressive behavior of this renal malignancy and summarized its therapeutic modalities and outcomes.

**Conclusion:**

The diagnosis of RES relies on integrated analysis including histomorphology, immunohistochemical staining and confirmation of molecular-genetic testing. Despite the surgery and adjuvant therapy, optimized and potent therapeutic regimes are still urgently needed to improve the poor prognosis of RES.

## Background

Ewing sarcoma (ES), also known as primitive neuroectodermal tumors (PNET), is a group of undifferentiated tumors that originates from neuroectoderm. It typically encountered in the bone and soft tissue of children and young adults [1]. The occurrence of ES/PNET in kidney is firstly depicted in 1975 [2]. Up to date, there emerges limited reported cases of ES/PNET in kidney by the rarity of this disease. Renal Ewing sarcoma (RES) presents highly malignant, grows rapidly, and metastases early to the lung, bone and lymph node [3, 4]. Thus, it is indispensable to distinguish RES from other renal entities. Herein, we present a case of an adult patient with RES who was diagnosed and administered in our hospital. We report this case in accordance with the CARE-Guideline [5].

## Case presentation

**Fig. 1:**
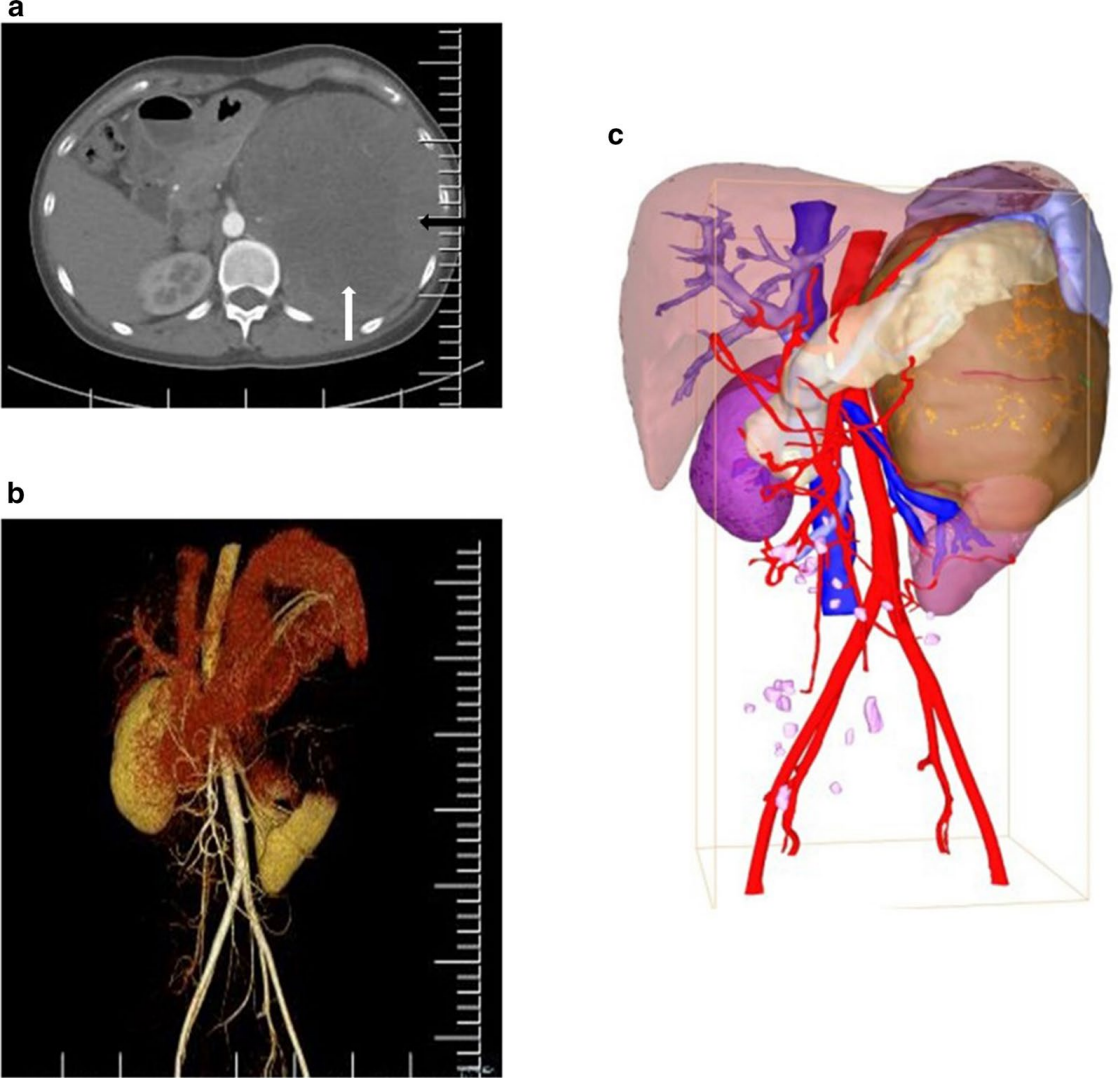
CT scan and three-dimensional imaging. Enhanced computed tomography (CT) revealed a hypoechoic mass about 18 cm × 14.5 cm × 14 cm arising from the left renal. **a** Cross-section. The black arrows represent the enhanced solid tumors and the white arrows represent non-enhanced tumor necrosis components. **b** Angiography of bilateral renal. **c** Three-dimensional imaging of the neoplasm

**Fig. 2:**
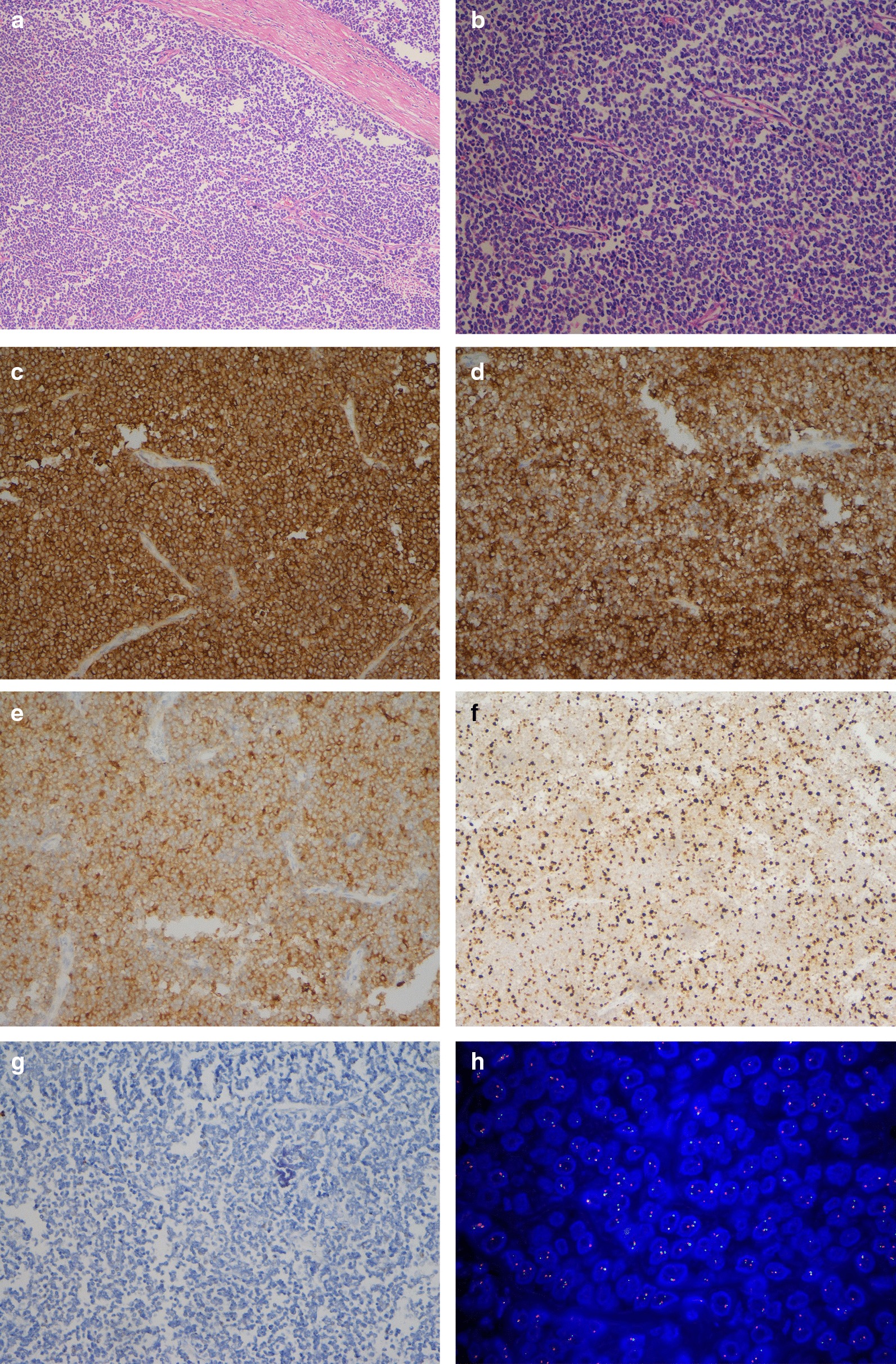
Pathological examination of the surgical specimen. HE staining (**a** and **b**) indicated that the tumor was composed of a population of small round cells arranged in sheet. Immunohistochemical staining was positive for **c** CD99, **d** CD56, **e** syn and **f** Ki67 (50%) and negative for **g** S100. Magnification details: **a** × 100; **b** × 200; **c** × 100; **d** × 100; **e** × 200; **f** × 100; **g** × 100. **g** EWSR1 gene translocation was detected by fluorescence in situ hybridization testing (FISH)

A 31-year-old female patient was admitted with chief complaints of intermittent pain in the left plank and palpable abdominal mass. No urinary symptoms such as haematuria and dysuria were found. Her past medical history and family history of malignancy were unremarkable. Results of routine blood and urine examination as well as tumor markers including AFP, AFP-L3, CEA, CA199, CA125 and SCC were within the normal range. Enhanced computed tomography (CT) demonstrated a hypoechoic mass (18 cm × 14.5 cm × 14 cm) from the left renal with central necrosis, no invasion to renal veins or inferior vena cava (IVC) was reported (Fig. 1a, b). Further clinical investigations including chest HRCT showed no evidence of metastasis. 3-dimensional imaging of the neoplasm was conducted (Fig. 1c), and an open, left radical nephrectomy was performed. Macroscopic examination showed a huge soft tissue mass attached to the spleen and pancreas tail. In surgery, the spleen was separated from the tumor capsule, and partial left adrenal gland and distal pancreatic involving about 2 cm × 1.5 cm × 1 cm pancreas tail tissue were excised with the assistance of hepatobiliary surgeons. Microscopic examination revealed a small round cell tumor with focal necrosis, endovascular tumor thrombi and adrenal involvement were detected (Fig. 2a, b). The tumor cells were arranged in sheet with fiber segmentation. Immunohistochemistry results indicated positive stained for AE1/AE3, CD99, CD56, synaptophysin (Syn), Ki-67(50%), and negatively stained for S-100, EMA, CgA, WT1, Desmin, SMA and MyoD1 (Fig. 2c–g). In the light of these findings, a pathological diagnosis of primary ES/PNET of the left renal was made. The diagnosis was further confirmed by fluorescence in situ hybridization (FISH) by detecting the rearrangements of t (22q12): EWS-FLI1 type 1 translocation (Fig. 2h). A PET-CT examination was conducted at 3 months postoperatively and showed no recurrence or metastasis. Adjuvant chemotherapy including vincristine, doxorubicin, cyclophosphamide (VAC), ifosfamide, etoposide (IE) and carboplatin (AUC = 4) was delivered after surgery. During the triweekly chemotherapy, metastasis was found in the retroperitoneal lymph node at 6 months postoperative, followed by multiple metastases to the left neck, bilateral adrenal gland, psoas major, retroperitoneum and bilateral diaphragm angles. The chemotherapy was maintained by the follow-up at 12 months postoperatively, and lung metastasis was detected by PET-CT scan. Afterwards, the antiangiogenic drug, apatinib, was recommended by the oncologist due to the intolerance to chemotherapy. This patient survived with tumor by the latest follow-up at 18 months postoperatively.

## Discussion

ES rarely presents as a primary kidney tumor. The clinical symptoms and signs are usually nonspecific, such as abdominal or flank pain, hematuria and palpable mass. Imaging examinations often reveal indistinctive signs but central necrosis and hemorrhage, which raises the difficulty of accurate preoperative diagnosis of RES. Mostly, the diagnosis was made by postoperative pathological results. Histologically, RES is composed of uniform small round cells. The major differential diagnosis for this histomorphology in kidney consists of Wilms tumor, malignant lymphoma, synovial sarcoma, solid variant of alveolar rhabdomyosarcoma, clear cell sarcoma of the kidney, small-cell neuroendocrine carcinoma, desmoplastic small round blue cell tumor and small cell carcinoma [6]. These poorly differentiated small round cell tumors share with overlapping morphologic features but have varied prognosis, which makes the distinguish diagnosis notoriously difficult but imperative. Advances in immunohistochemistry and molecular-genetic techniques are helpful, as numerous positive and negative immunohistochemical markers offer a better distinction. CD99 and FLI-1 were commonly positive in ES/PNET, including those arising from the kidney. CD45, WT-1 and Desmin have been reported as negative [7, 8]. These markers by immunohistochemical staining established a useful albeit imprecise diagnosis panel of RES, as some of the markers are shared among small round blue tumors. For the indefinable cases, the ancillary molecular testing is recommended [9, 10]. ES/PNET is characterized by fixed chromosomal translocation t (11:22) between the genes EWS (22q12) and FLI-1 (11q24). In the present case, the EWS-FLI1 type 1 translocation was detected by FISH.

In view of the striking rarity of RES, the standard treatment for this tumor remains absence and mostly referred to clinical regimens of osseous Ewing’s sarcoma (ESB). Currently, radical nephrectomy followed by chemotherapy was commendatory. Surgical intervention may incorporate with cavotomy in the presence of venous tumor thrombus, which was frequently reported in RES and was stated to be associated with the presence of pulmonary metastasis [11, 12]. Chemotherapy was recognized to be beneficial to RES survival [13]. The commonly used chemotherapeutic regimes are comprised of multiple agents including vincristine, ifosfamide, doxorubicin, etoposide, cyclophosphamide, etoposide or actinomycin D. In the latest years, neoadjuvant chemotherapy following biopsy was acceptable [14], however, its survival benefits need to be verified in the future. Radiation has shown some success as salvage therapy for targeted lesions. However, conflicting views exist surrounding recommending radiotherapy as the primary treatment modality [15]. Given the multiple metastasis and the poor physical condition of this patient, radiation therapy was not recommended by oncologists. Owing to the aggressive adjuvant therapy, the prognosis of localized RES is improved [16], whereas the survival of patients with metastasis at initial diagnosis remains poor [17]. In addition, the newly approved anti-tumor agents, such as the antiangiogenic drug apatinib [18], were claimed to serve as one of the therapeutic options for RES. More studies are needed to validate the benefits of targeted anti-tumor agents including apatinib on the recurrence free and overall survival of RES patient in the future.

Due to its atypical location as stated, the diagnosis of ES/PNET in kidney relies on integrated analysis including histomorphology, multiple immunohistochemical staining and confirmation of molecular-genetic testing. Despite the improved prognosis of this tumor by surgery and adjuvant therapy, optimized and potent therapeutic regimes are still urgently needed.

## Data Availability

All data and materials in the manuscript will be available from the corresponding author for non-commercial purposes.

## Abbreviations

ES: Ewing sarcoma
PNET: Primitive neuroectodermal tumors
RES: Renal Ewing sarcoma
CT: Computed tomography
IVC: Inferior vena cava
PET-CT: Positron emission tomography-computed tomography
VAC: Vincristine, doxorubicin and cyclophosphamide
IE: Ifosfamide and etoposide
FISH: Fluorescence in situ hybridization testing
